# Differentiating linked cognitive states under task demand and sleep restriction: toward an experimental framework for operational monitoring

**DOI:** 10.3389/fnrgo.2026.1909914

**Published:** 2026-10-06

**Authors:** Maria Rivas-Vidal, Alberto Calvo Cordoba, Fernando Daniel Farfán, Cecilia E. García-Cena

**Affiliations:** 1 Escuela Técnica Superior de Ingenieros Industriales, Universidad Politécnica de Madrid, Madrid, Spain; 2 Centre for Automation and Robotics (UPM-CSIC), Escuela Técnica Superior de Ingeniería y Diseño Industrial, Universidad Politécnica de Madrid, Madrid, Spain; 3 Indra Sistemas, S.A., Madrid, Spain; 4 Institute of Bioengineering, Universidad Miguel Hernández of Elche, Elche, Alicante, Spain; 5 Laboratorio de Investigación en Neurociencias y Tecnologías Aplicadas (LINTEC), Departamento de Bioingeniería, Facultad de Ciencias Exactas y Tecnología, Universidad Nacional de Tucumán, San Miguel de Tucumán, Tucumán, Argentina; 6 Instituto Superior de Investigaciones Biológicas (INSIBIO), Consejo Nacional de Investigaciones Científicas y Técnicas (CONICET), San Miguel de Tucumán, Tucumán, Argentina

**Keywords:** drowsiness, mental fatigue, mental workload, mind wandering, behavioral measures, subjective measures

## Abstract

Real-world settings involve multiple interacting cognitive states such as mental workload, drowsiness, fatigue, and mind wandering, yet these are typically studied in isolation. A critical question is whether these states can be empirically differentiated given that they share overlapping behavioral and physiological signatures. We investigated whether task difficulty and sleep restriction produce dissociable effects across these states, and whether their response patterns reflect distinct underlying mechanisms or a common cognitive resource using a unified framework. Participants from operational populations (Study 1: *n* = 40; Study 2: *n* = 27) performed three complementary tasks (MATB-II, N-Back, and SART) under two manipulations: task difficulty and partial sleep restriction, targeting cognitive load and alertness. Increasing task difficulty degraded performance and increased perceived workload and fatigue across tasks. In contrast, sleep restriction primarily elevated subjective drowsiness and mind wandering, with smaller, task-dependent effects on performance. These findings provide evidence that cognitive and physiological states influencing situational awareness can be differentiated using complementary manipulations and characterized through distinct behavioral and subjective measures. This distinction provides a foundation for future predictive and monitoring approaches in applied settings.

## 1 Introduction

Maintaining adequate situational awareness is critical in complex and dynamic operating environments, such as aviation, defense, and other high-stakes industries. In these settings, fluctuations in operator cognitive and attentional functioning can significantly impact human performance and safety (Mohammadfam et al., 2021; Naderpour et al., 2015). Cognitive states such as mental workload, fatigue, drowsiness, and mind wandering reflect different aspects of an operator's cognitive functioning, including the demands imposed by a task, changes in alertness, and variations in sustained attention and attentional engagement. Therefore, understanding how such states emerge and interact is essential for developing reliable systems capable of monitoring and supporting highly demanding processes in real-time (Endsley, 2001; Graafland et al., 2015; Rodriguez et al., 2017).

Research has traditionally examined cognitive states such as mental workload, fatigue, drowsiness, and mind wandering in isolation. While this approach has clarified individual mechanisms, it does not reflect real-world conditions, where these states often co-occur or evolve dynamically. For instance, sustained workload may lead to fatigue (Borghini et al., 2014; Chowdhury et al., 2018; Freitas et al., 2024), while sleep-related factors contribute to drowsiness and fatigue (Borghini et al., 2014; Chowdhury et al., 2018; Freitas et al., 2024; Shen et al., 2006), and both can influence the occurrence of mind wandering (Zanesco et al., 2025). Despite these well-established relationships, few studies have investigated these states within a unified experimental framework. As an example, Hanzal and coworkers investigated both fatigue and mind wandering, finding subjective convergence (Hanzal et al., 2026).

A wide range of paradigms and measurement techniques have been used to study these states, including task difficulty manipulations, multitask environments, vigilance tasks, and sustained attention paradigms (Chu et al., 2023; Longo et al., 2022; Pontiggia et al., 2024; Shahid et al., 2010; Shen et al., 2006). Assessment approaches typically combine subjective reports, performance-based measures, and physiological indicators. Subjective measures include widely used scales such as NASA Task Load Index (NASA-TLX), Karolinska Sleepiness Scale (KSS), or Visual Analog Scales (VAS), while performance metrics typically rely on accuracy, reaction time, and response variability (Chu et al., 2023; Ebrahim Shaik, 2023; Hawkins et al., 2015; Karim et al., 2024; Kunasegaran et al., 2023; Longo et al., 2022; Young et al., 2015). Physiological indicators encompass ocular, cardiac, electrodermal, respiratory, and brain activity signals. However, many of these states share overlapping signatures. For instance, increases in reaction time have been associated with several of the target conditions, including drowsiness, fatigue, and mind wandering (Chowdhury et al., 2018; Karim et al., 2024; Nazari et al., 2025; Pan et al., 2024). Variations in ocular behavior, such as reduced pupil diameter and increased blink frequency, have been reported in fatigue, drowsiness, mental workload, and mind wandering (Bafna and Hansen, 2021; Charles and Nixon, 2019; Chowdhury et al., 2018; Hamann et al., 2026; Hu and Lodewijks, 2020; Kolus, 2024; Longo et al., 2022; Nazari et al., 2025; Pan et al., 2024). Similarly, changes in alpha and theta brain activity have also been associated with fatigue, drowsiness, mental workload, and mind wandering (Charles and Nixon, 2019; Chowdhury et al., 2018; Hamann et al., 2026; Hu and Lodewijks, 2020; Karim et al., 2024; Longo et al., 2022; Nazari et al., 2025; Sahayadhas et al., 2012). This overlap complicates the differentiation of cognitive states, particularly in applied settings.

In this context, the present work investigates whether multiple related operator cognitive states (mental workload, drowsiness, mental fatigue, and mind wandering) can be jointly elicited and differentiated within a controlled setting. To this end, we introduce a multimodal dataset integrating performance, subjective, and physiological recordings collected during three complementary tasks: Multi-Attribute Task Battery II (MATB-II), N-Back, and Sustained Attention to Response Task (SART). The dataset combines two experimental protocols: Study 1 manipulates two levels of task difficulty, while Study 2 compares performance before and after a 2-h sleep period (partial sleep restriction). These manipulations target complementary mechanisms, namely increased cognitive load and reduced alertness. Statistical analyses of performance metrics and subjective questionnaires provide an initial validation of the dataset, revealing robust effects of task difficulty and more selective, primarily subjective effects of partial sleep restriction. These results support the feasibility of capturing interacting and partially overlapping cognitive states within a unified experimental setting, providing a basis for future multimodal modeling and physiological signal analysis.

## 2 Materials and methods

### 2.1 Participants

The sample for each of the two studies consisted of 40 (29 men, 11 women; aged 25 to 45, mean 33.1± 6.2 years) and 27 (20 men, 7 women; aged 19 to 43, mean 30.9 ± 5.1 years) healthy adult participants, respectively. For Study 1 most participants were firefighters (*n* = 17), followed by police officers (*n* = 15), military personnel (*n* = 7), and one other profession. For Study 2, professions included police officers (*n* = 10), military personnel (*n* = 8), firefighters (*n* = 6), and others (*n* = 3).

The studies did not necessarily share participants, although individuals were informed about both studies and given the option to participate in one or both. Participants were recruited via public advertisements (e.g., online platforms, flyers) and through an existing volunteer database. Additionally, they received financial compensation for their participation (75€ in Study 1 and 100€ in Study 2).

Participants were eligible for inclusion if they were between 25 and 45 years of age and had sufficient knowledge of Spanish to understand the consent form and task instructions. One participant in Study 2 was 19 years old and therefore fell outside the predefined eligibility range, but was retained in the study. Exclusion criteria included serious medical treatments or medications that could interfere with results, history of neurological or psychiatric disorders, significant health problems in the previous 12 months (particularly neurological or cardiac), pregnancy or breastfeeding, and the use of psychotropic medication, benzodiazepines, or gamma-hydroxybutyric acid during the study period.

### 2.2 Equipment

Participants sat in a chair facing a 24-inch computer monitor positioned approximately 60 cm from their eyes. Before sensor placement, a brief familiarization phase was conducted for each task to ensure that initial performance differences due to novelty effects (influence on task execution as a response to the newness of an activity) were minimized. In second-session visits, familiarization served as a brief reminder of task procedures.

Physiological signals were recorded using non-invasive wearable sensors. Electroencephalography (EEG) activity was recorded with the 32-channel Bitbrain Versatile + system (32 channels). Electrodermal activity (EDA), electrocardiography (ECG), blood pulse volume (BPV), and respiratory signals were collected with the Bitbrain Versatile Bio device. Both devices were sampled at 256 Hz. Eye movements and pupil diameter were monitored using a Tobii Pro Glasses 3 eye-tracker operating at 50 Hz. Any problems detected during recording were documented by the laboratory team. The analysis of these physiological signals is not within the scope of this article, which focuses exclusively on behavioral and subjective measures.

### 2.3 Experimental design

This research consisted of two independent studies designed to evaluate the feasibility of eliciting and characterizing related operator cognitive and physiological states. Both studies employed the same experimental tasks to induce and assess changes in cognitive state but differed in the configuration used to trigger the intended changes.

#### 2.3.1 General procedure

Each recording session began with two baseline recordings: 3 min with eyes open while fixating on a cross displayed on a computer screen, followed by 3 min with eyes closed. Participants then performed three cognitive tasks presented in randomized order: N-Back, SART, and MATB-II. The N-Back test required participants to indicate whether the digit presented on the screen matched the one shown *N* steps earlier in the sequence, with task difficulty manipulated by adjusting the value of *N*. The SART task involved a rapid presentation of digits (1 through 9) on the screen. Participants were instructed to press a key for each digit except when a specific one (number 3) appeared, requiring sustained inhibition of response. Lastly, the MATB-II task (Comstock and Arnegard, 1992) is a computer-based test designed to simulate multitasking operational environments, particularly those faced by an aircraft crew during flight. Four subtasks are displayed and must be managed simultaneously on the same screen. After completing the cognitive tasks, participants repeated the baseline recordings. The total duration of each session was approximately 90 min, including sensor placement and removal (Figure 1).

**Figure 1:**
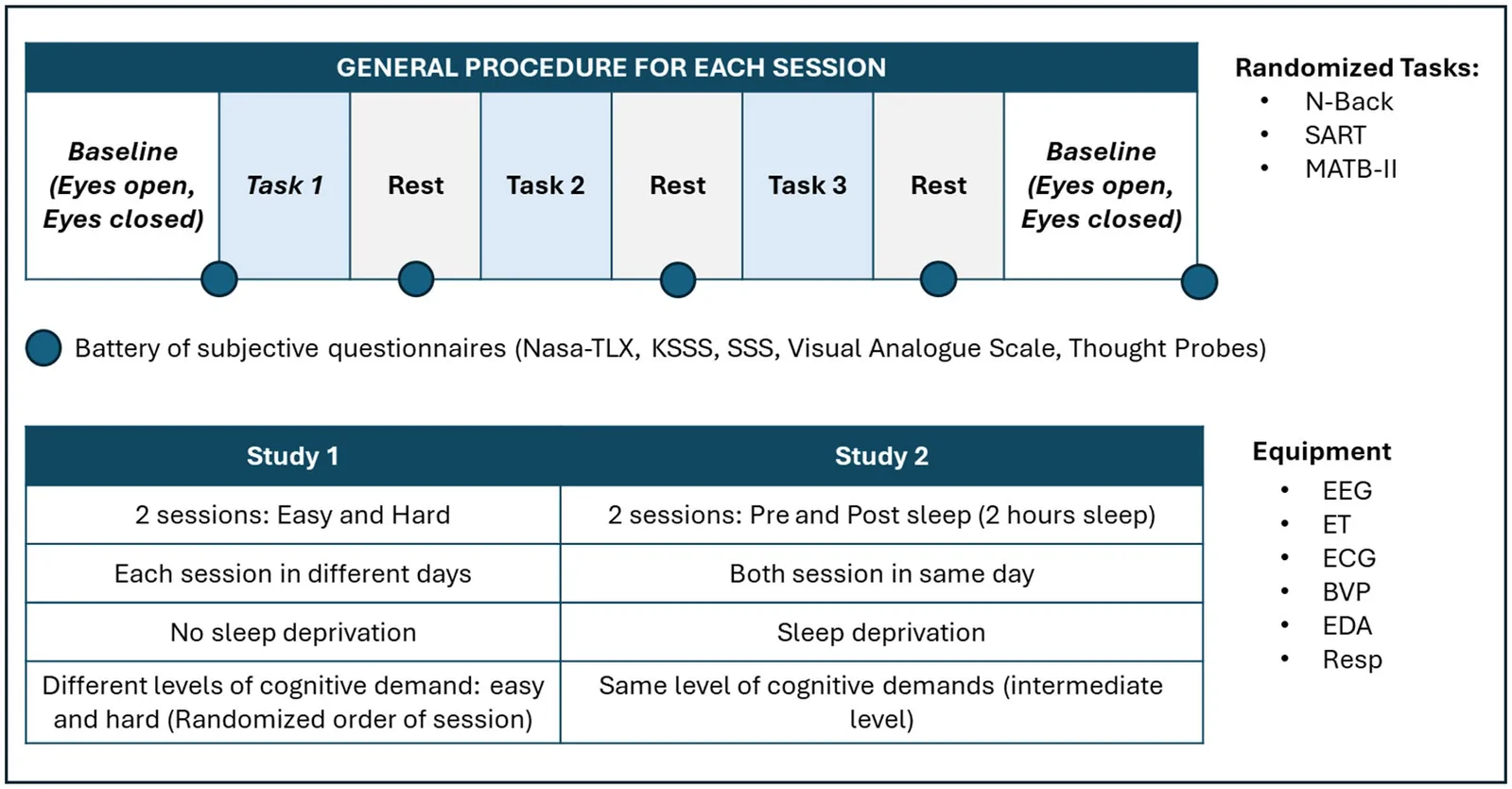
General procedure followed for each of the sessions and details of each of the conducted studies. NASA-TLX, NASA Task Load Index; KSS, Karolinska Sleepiness Scale; SSS, Stanford Sleepiness Scale; VAS, Visual Analog Scale; SART, Sustained Attention to Response Task; MATB-II, Multi-Attribute Task Battery II; EEG, electroencephalography; ET, eye-tracking; ECG, electrocardiography; BPV, blood pulse volume; EDA, electrodermal activity; Resp, respiration.

Additionally, following each baseline and task, participants completed a series of subjective questionnaires that assessed perceived cognitive demand, self-rated attention, and drowsiness levels. This battery of questionnaires included the NASA-TLX, KSS, SSS, VAS, and thought probes for mind wandering. The NASA-TLX questions assessed perceived workload across six dimensions: mental demand, physical demand, temporal demand, perceived performance, effort, and frustration, each rated on a −10 to 10 scale, with higher scores indicating greater perceived demand, effort, frustration, or better perceived performance, as appropriate. The KSS and SSS assessed subjective sleepiness using nine- and seven-point response categories, respectively. The VAS assessed perceived sleepiness on a continuous 0–10 scale ranging from very alert to having fallen asleep. The thought probes assessed (1) the content of participants' thoughts during the task, using six response categories ranging from task-focused thoughts to task-unrelated thoughts or lack of a specific thought, (2) the perceived difficulty of disengaging from an off-task thought and returning to the task, using a 5-point scale, and (3) perceived attention during the task on a 0–10 scale ranging from very inattentive to fully focused.

To provide a clear overview of how the experimental tasks and measurements relate to the target conditions, a conceptual mapping was established between each condition, the tasks employed to elicit it, the physiological signals recorded, and the subjective questionnaires administered (Table 1).

**Table 1:** Conceptual mapping between target conditions and their associated experimental tasks, subjective questionnaires, and physiological signals.

Condition	Experimental tasks	Subjective questionnaires	Physiological signals
Mental workload	N-Back, MATB-II (Study 1)	NASA-TLX	EEG, ET, ECG, EDA, Resp
Drowsiness	MATB-II, N-Back, SART (Study 2)	KSS, SSS, VAS	EEG, ET, ECG, EDA
Mental fatigue	MATB-II, N-Back, SART (Study 1 and Study 2)	VAS, KSS, SSS, NASA-TLX	EEG, ET, ECG, EDA
Mind wandering	SART, MATB-II (Study 1)	Thought probes	EEG, ET

NASA-TLX, NASA Task Load Index; KSS, Karolinska Sleepiness Scale; SSS, Stanford Sleepiness Scale; VAS, Visual Analog Scale; SART, Sustained Attention to Response Task; MATB-II, Multi-Attribute Task Battery II; EEG, electroencephalography; ET, eye-tracking; ECG, electrocardiography; EDA, electrodermal activity; Resp, respiration.

#### 2.3.2 Study 1: task difficulty manipulation

Study 1 consisted of two sessions separated by no more than 14 days. Both sessions involved the same task sequence described in the general procedure, but the level of task difficulty was systematically manipulated (easy and hard levels). The different level configurations were determined by varying parameters such as presentation time, inter-stimulus interval, response interval, number of trials, or characteristics specific to the nature of the task to be performed. The order of sessions was randomized across participants to account for order effects.

#### 2.3.3 Study 2: partial sleep restriction

Study 2 investigated the impact of partial sleep restriction. This study also involved two sessions following the general procedure, but both were conducted on the same day and used the same level of difficulty. The first session took place approximately 2 h before each participant's typical bedtime. After completion, participants were prepared for an overnight stay at the research facility. After approximately 2 h of sleep, participants were awakened and immediately performed the second session under identical task conditions.

### 2.4 Database structure

All data were organized by study, session, and participant to ensure consistency. Each dataset included synchronized physiological, behavioral, and metadata files, structured according to the experimental tasks. Physiological signals are stored alongside timestamped behavioral and interaction data, allowing for multimodal alignment.

### 2.5 Data processing

#### 2.5.1 Performance metrics derivation

For performance metrics, an initial set of indicators was extracted for each task (24 for MATB-II, 16 for N-Back, and 16 for SART). Mean measures were retained over corresponding median measures, while derived or component metrics providing overlapping information were excluded (e.g., sum-of-squares). Auxiliary measures not describing performance itself (e.g., event counts) were also excluded. This resulted in a final subset of six representative metrics per task.

MATB-II task is composed of four subtasks, each with respective performance metrics. For the Tracking task, participants had to keep the target in the center of the grid using a joystick. Performance was quantified using the root mean square deviation (RMSD) between the target trajectory and the participant-controlled cursor (joystick), computed across all valid samples within each session. RMSD values were averaged at the session level for each participant. For Resource Management (RMAN) task, participants had to maintain the volume of fuel in the tanks within the specified range. Performance was indexed by the mean absolute deviation between required and actual fuel tank levels. Deviations were computed for all valid samples and averaged per session, with higher values indicating poorer resource regulation. For the Communications (Comm) task, participants had to listen to radio messages and respond only to those addressed to their call sign (do as instructed). Two complementary measures were derived: mean reaction time (RT) and response accuracy. RTs were calculated as the mean response latency for all valid responses (RT > 0) within a session. Accuracy was defined as the proportion of correctly handled communication events, with partially correct responses excluded from accuracy calculations but retained for descriptive inspection. For System Monitoring (SysMon), participants had to monitor lights and dial oscillations in order to intervene if any element deviated from their normal state (system failure). A light failure occurs when the green light turns off or the red light turns on. On the other hand, a dial failure occurs when they stop oscillating around the center of the scale. Performance was similarly assessed using mean RT and accuracy. RTs were computed only for failure events requiring an explicit participant response, while accuracy was defined as the proportion of correctly identified system failures relative to the total number of failure events. Background system state updates not requiring a response were excluded from RT calculations.

Performance in the N-back task was assessed based on trial-level response classifications. Each trial was classified as a true positive (TP), true negative (TN), false positive (FP), or false negative (FN) according to the correspondence between stimulus type and participant response. Responses to target stimuli were classified as true positives (responding when should respond), whereas correct withholding of responses to non-target stimuli was classified as true negatives (not responding when shouldn't respond). Incorrect responses and missed targets were classified as false positives (responding when shouldn't respond) and false negatives (not responding when should respond), respectively. From this classification, accuracy was computed as the proportion of correct trials (TP + TN) relative to the total number of trials. Error rate was defined as the proportion of incorrect trials (FP + FN) with respect to the total number. The F1 score was calculated as 2 × TP/(2 × TP + FP + FN), providing a summary measure that reflects the ability to identify targets while accounting for both missed targets and incorrect responses to non-target stimuli. RT metrics were computed using valid numeric RTs only and RT variability was defined as the standard deviation of RTs within the session. Lastly, a timeout was operationally defined as a false negative trial, corresponding to a failure to respond when a response was required. The timeout rate was computed as the proportion of timeout trials relative to the total number of trials.

Lastly, the SART task performance was analyzed using the same trial-level response classification and metric definitions as described for the N-back task, with task-specific adaptations to the response rules. In the SART, correct withholding of responses to no-go stimuli was classified as a true positive (not responding when shouldn't respond), while correct responses to go stimuli were classified as true negatives (responding when should respond). All other response patterns were classified as false positives (not responding when should respond) or false negatives (responding when shouldn't respond) accordingly.

#### 2.5.2 Subjective condition scores derivation

Composite scores were computed for each target condition by aggregating relevant questionnaire items associated with each target construct. The choice of tasks for each target condition was based on the study design (Table 1). Consequently, not all tasks were included in the derivation of every subjective score.

Mental workload was derived from NASA-TLX dimensions mental demand, temporal demand, and effort; drowsiness from KSS, SSS, and VAS items; fatigue from all six NASA-TLX dimensions combined with the three drowsiness-related items (to capture a broad subjective fatigue construct reflecting both perceived task demands and sleepiness-related influences); and mind wandering from thought probe responses.

All items were rescaled to a 0–1 range using min–max normalization based on the theoretical bounds of each question (e.g., NASA-TLX: −10 to 10, KSS: 1–9). Items where higher values reflected more favorable states were reverse-scored to ensure that higher normalized values consistently reflected higher levels of the target construct.

#### 2.5.3 Statistical analysis

Derived performance metrics and subjective scores were analyzed to evaluate whether the experimental manipulations induced the intended operator cognitive and physiological states. Analyses focused on within-participant session comparisons (*Easy* vs. *Hard* in Study 1; *Pre-Sleep* vs. *Post-Sleep* in Study 2), as well as task-level differences where appropriate. Normality of each metric was assessed using the Shapiro-Wilk test. When normality assumptions were met, parametric tests were applied (paired-sample or independent-sample *t*-tests, depending on design); otherwise, non-parametric alternatives were used (Wilcoxon signed-rank or Mann-Whitney *U* tests). Effect sizes were reported as Cohen's *d* for parametric tests and rank-biserial *r* for non-parametric tests. Additionally, they were interpreted according to conventional benchmarks (Cohen, 1977a,b) based on their absolute magnitude, while the sign indicates the direction of the effect: negligible (|*d*| < 0.2; |*r*| < 0.1), small (0.2 ≤ |*d*| < 0.5; 0.1 ≤ |*r*| < 0.3), medium (0.5 ≤ |*d*| < 0.8; 0.3 ≤ |*r*| < 0.5), and large (|*d*| ≥ 0.8; |*r*| ≥ 0.5). To control for multiple comparisons across metrics, *p*-values were adjusted using the Holm correction.

### 2.6 Ethics statement

This research complied with the Declaration of Helsinki and was approved by the Institutional Review Board at *Comité de Ética de la Investigación de la Comunidad Autónoma de Aragón* (CEICA), approval code PI24/356. Informed consent was obtained from each participant, who received oral and written information about the study, its purpose, potential risks, and inclusion and exclusion criteria.

## 3 Results

### 3.1 Performance metrics analysis

#### 3.1.1 MATB-II

**Study 1**. Performance differed significantly between *Easy* and *Hard* sessions (Table 2). Tracking RMSD, RMAN deviations, and SysMon mean RT increased in *Hard* sessions (all with medium to large effect sizes), while Comm mean RT and accuracy decreased (large and small effect sizes, respectively). SysMon accuracy increased slightly but did not differ significantly (small effect size).

**Table 2:** MATB-II metrics statistics.

Study 1 (hard vs. easy sessions)						
**Metric**	**Easy (M** ±**SD)**	**Hard (M** ±**SD)**	**% Change**	**Test**	***p*** **(Holm)**	**Effect size**
Tracking RMSD	30.54 ± 9.44	36.51 ± 5.58	↑ 19.5%	Paired t-test	0.003^**^	*d* = 0.56 (medium)
RMAN deviation	247.88 ± 116.57	341.11 ± 144.98	↑ 37.6%	Paired t-test	< 0.001^***^	*d* = 0.76 (medium)
Comm mean RT (s)	20.08 ± 1.29	6.49 ± 1.24	↓ 67.7%	Paired t-test	< 0.001^***^	*d* = −9.66 (large)
Comm accuracy	0.89 ± 0.14	0.83 ± 0.16	↓ 7.6%	Paired t-test	0.038^*^	*d* = −0.39 (small)
SysMon mean RT (s)	3.21 ± 1.21	4.31 ± 0.75	↑ 34.3%	Paired t-test	< 0.001^***^	*d* = 0.88 (large)
SysMon accuracy	0.70 ± 0.37	0.77 ± 0.24	↑ 9.6%	Wilcoxon	0.189	*r* = 0.28 (small)
**Study 2 (post-sleep vs. pre-sleep sessions)**						
**Metric**	**Pre-sleep (M** ±**SD)**	**Post-sleep (M** ±**SD)**	**% Change**	**Test**	***p*** **(Holm)**	**Effect size**
Tracking RMSD	39.24 ± 5.02	36.03 ± 4.57	↓ 8.2%	Paired t-test	0.024^*^	*d* = −0.62 (medium)
RMAN deviation	550.93 ± 321.06	479.91 ± 329.27	↓ 12.9%	Wilcoxon	0.179	*r* = 0.35 (medium)
Comm mean RT (s)	7.03 ± 1.65	6.19 ± 1.37	↓ 11.9%	Paired t-test	0.022^*^	*d* = −0.65 (medium)
Comm accuracy	0.80 ± 0.16	0.90 ± 0.10	↑ 13.1%	Paired t-test	0.036^*^	*d* = 0.56 (medium)
SysMon mean RT (s)	5.11 ± 1.02	4.48 ± 0.74	↓ 12.3%	Paired t-test	0.009^**^	*d* = −0.73 (medium)
SysMon accuracy	0.89 ± 0.16	0.91 ± 0.13	↑ 2.4%	Wilcoxon	0.322	*r* = 0.37 (medium)

↑/↓ indicates direction of change. Significance: ^*^p < 0.05, ^**^p < 0.01, ^***^p < 0.001.

MATB-II, Multi-Attribute Task Battery II; RMSD, root mean square deviation; RMAN, resource management; Comm, communications; RT, reaction time; SysMon, system monitoring.

**Study 2**. Similarly, several metrics showed differences from *Pre-Sleep* to *Post-Sleep* session (Table 2). Comm accuracy increased while Comm mean RT, SysMon mean RT, and tracking RMSD were lower in *Post*-*Sleep* session compared to *Pre-Sleep* (medium effect sizes). No significant changes were found for RMAN deviation or SysMon accuracy (both with medium effect size), though descriptive trends suggested a slight increase.

#### 3.1.2 N-back

**Study 1**. N-Back performance metrics were significantly affected by session, all with large effect sizes (Table 3). Accuracy and F1 score decreased from *Easy* to *Hard*. Conversely, mean RT, RT variability, error rate and timeout rate increased during *Hard* sessions compared to *Easy* sessions.

**Table 3:** N-back metrics statistics.

Study 1 (hard vs. easy sessions)						
**Metric**	**Easy (M** ±**SD)**	**Hard (M** ±**SD)**	**% Change**	**Test**	***p*** **(Holm)**	**Effect size**
Accuracy	0.99 ± 0.01	0.93 ± 0.04	↓ 5.7%	Wilcoxon	< 0.001^***^	*r* = 0.87 (large)
F1 score	0.98 ± 0.07	0.86 ± 0.09	↓ 12.2%	Wilcoxon	< 0.001^***^	*r* = 0.87 (large)
Mean RT (s)	0.63 ± 0.15	0.70 ± 0.12	↑ 10.8%	Wilcoxon	< 0.001^***^	*r* = 0.52 (large)
RT variability	0.16 ± 0.07	0.25 ± 0.07	↑ 60.0%	Wilcoxon	< 0.001^***^	*r* = 0.74 (large)
Error rate	0.01 ± 0.01	0.07 ± 0.04	↑ 582.0%	Wilcoxon	< 0.001^***^	*r* = 0.90 (large)
Timeout rate	0.01 ± 0.01	0.04 ± 0.03	↑ 480.9%	Wilcoxon	< 0.001^***^	*r* = 0.86 (large)
Study 2 (post-sleep vs. pre-sleep sessions)						
**Metric**	**Pre-sleep (M** ±**SD)**	**Post-sleep (M** ±**SD)**	**% change**	**Test**	***p*** **(Holm)**	**Effect size**
Accuracy	0.93 ± 0.04	0.92 ± 0.05	↓ 0.3%	Paired *t*-test	1.00	*d* = −0.11 (negligible)
F1 score	0.84 ± 0.08	0.84 ± 0.10	↓ 0.6%	Wilcoxon	1.00	*r* = 0.10 (negligible)
Mean RT (s)	0.68 ± 0.13	0.70 ± 0.14	↑ 2.2%	Paired *t*-test	1.00	*d* = 0.21 (small)
RT variability	0.24 ± 0.08	0.25 ± 0.07	↑ 1.8%	Paired *t*-test	1.00	*d* = 0.08 (negligible)
Error rate	0.07 ± 0.04	0.08 ± 0.05	↑ 3.7%	Paired *t*-test	1.00	*d* = 0.11 (negligible)
Timeout rate	0.05 ± 0.03	0.05 ± 0.03	↑ 5.1%	Paired *t*-test	1.00	*d* = 0.10 (negligible)

↑/↓ indicates direction of change. Significance: ^*^p < 0.05, ^**^p < 0.01, ^***^p < 0.001. RT, reaction time.

**Study 2**. For Study 2, no significant changes were observed in N-Back performance between *Pre-Sleep* and *Post-Sleep* sessions (Table 3). Accuracy and F1 score remained stable (slight decrease, negligible effect sizes). Mean RT, RT variability, error rate and timeout rate increased, but showed no statistically significant change (all with negligible or small effect sizes).

#### 3.1.3 SART

**Study 1**. SART performance differences between *Easy* and *Hard* sessions showed mixed results (Table 4). Error and timeout rates increased in the *Hard* session, both reaching statistical significance (large effect sizes). Accuracy, F1 score, and mean RT decreased significantly (all with large effect sizes), while RT variability decreased slightly but did not reach significance (small effect size).

**Table 4:** SART metrics statistics.

Study 1 (hard vs easy sessions)						
**Metric**	**Easy (M** ±**SD)**	**Hard (M** ±**SD)**	**% Change**	**Test**	***p*** **(Holm)**	**Effect size**
Accuracy	0.99 ± 0.01	0.95 ± 0.03	↓ 4.1%	Paired *t*-test	< 0.001^***^	*d* = −1.49 (large)
F1 score	0.93 ± 0.07	0.70 ± 0.20	↓ 25.1%	Paired *t*-test	< 0.001^***^	*d* = −1.30 (large)
Mean RT (s)	0.53 ± 0.18	0.40 ± 0.08	↓ 23.5%	Wilcoxon	< 0.001^***^	*r* = 0.82 (large)
RT variability	0.11 ± 0.06	0.09 ± 0.03	↓ 14.3%	Wilcoxon	0.115	*r* = 0.25 (small)
Error rate	0.01 ± 0.01	0.05 ± 0.03	↑ 297.7%	Paired *t-*test	< 0.001^***^	*d* = 1.49 (large)
Timeout rate	0.01 ± 0.01	0.04 ± 0.03	↑ 275.6%	Paired *t-*test	< 0.001^***^	*d* = 1.40 (large)
Study 2 (post-sleep vs. pre-sleep sessions)						
**Metric**	**Pre-sleep (M** ±**SD)**	**Post-sleep (M** ±**SD)**	**% Change**	**Test**	***p*** **(Holm)**	**Effect size**
Accuracy	0.92 ± 0.04	0.93 ± 0.05	↑ 0.9%	Wilcoxon	0.506	*r* = 0.29 (small)
F1 score	0.56 ± 0.22	0.61 ± 0.27	↑ 8.2%	Paired *t*-test	0.506	*d* = 0.27 (small)
Mean RT (s)	0.37 ± 0.09	0.39 ± 0.11	↑ 4.5%	Paired *t*-test	0.506	*d* = 0.30 (small)
RT variability	0.10 ± 0.05	0.11 ± 0.07	↑ 10.6%	Paired *t*-test	0.192	*d* = 0.44 (small)
Error rate	0.08 ± 0.04	0.07 ± 0.05	↓ 10.3%	Wilcoxon	0.506	*r* = 0.30 (small)
Timeout rate	0.06 ± 0.03	0.05 ± 0.03	↓ 11.6%	Paired *t*-test	0.307	*d* = −0.38 (small)

↑/↓ indicates direction of change. Significance: ^*^p < 0.05, ^**^p < 0.01, ^***^p < 0.001. SART, Sustained Attention to Response Task; RT, reaction time.

**Study 2**. SART performance showed no significant changes from *Pre-Sleep* to *Post-Sleep* session (Table 4). Error and timeout rates increased in the *Post* session, while accuracy, F1 score, mean RT and RT variability decreased (all with small effect sizes).

### 3.2 Subjective questionnaires analysis

#### 3.2.1 Mental workload

**Study 1**. Both cognitive tasks, MATB-II and N-Back received significantly higher mental workload ratings compared to both pre and post baseline measurements (Table 5). Additionally, the *Hard* session was rated as more mentally demanding than the *Easy* session across all tasks. While baseline mental workload ratings showed non-significant increases in the *Hard* session moderate-to-large effect sizes were observed for both baseline comparisons. In contrast, both cognitive tasks demonstrated significant workload increases with large effect sizes, indicating higher perceived workload during the *Hard* condition.

**Table 5:** Subjective mental workload.

Study 1 (hard vs easy sessions)						
**Task**	**Easy (M** ±**SD)**	**Hard (M** ±**SD)**	**% Change**	**Test**	***p*** **(Holm)**	**Effect size**
Baseline 1 (pre)	0.06 ± 0.06	0.07 ± 0.08	↑ 12.4%	Wilcoxon	0.741	*r* = 0.47 (medium)
MATB-II	0.39 ± 0.25	0.64 ± 0.23	↑ 63.6%	Paired *t*-test	< 0.001^***^	*d* = 0.91 (large)
N-Back	0.33 ± 0.25	0.72 ± 0.21	↑ 120.2%	Paired *t*-test	< 0.001^***^	*d* = 1.48 (large)
Baseline 2 (post)	0.08 ± 0.13	0.12 ± 0.17	↑ 43.4%	Wilcoxon	0.139	*r* = 0.64 (large)
Study 2 (post-sleep vs. pre-sleep sessions)						
**Task**	**Pre-sleep (M** ±**SD)**	**Post-sleep (M** ±**SD)**	**% Change**	**Test**	***p*** **(Holm)**	**Effect size**
Baseline 1 (Pre)	0.20 ± 0.20	0.23 ± 0.21	↑ 13.4%	Paired *t*-test	1.000	*d* = 0.12 (negligible)
MATB-II	0.68 ± 0.14	0.57 ± 0.21	↓ 19.5%	Paired *t*-test	0.007^**^	*d* = −0.69 (medium)
N-back	0.81 ± 0.14	0.76 ± 0.14	↓ 6.4%	Paired *t*-test	0.176	*d* = −0.39 (small)
Baseline 2 (Post)	0.15 ± 0.18	0.24 ± 0.24	↑ 35.8%	Wilcoxon	0.176	*r* = 0.58 (large)

↑/↓ indicates direction of change. Significance: ^*^p < 0.05, ^**^p < 0.01, ^***^p < 0.001.

**Study 2**. Similarly to Study 1, both MATB-II and N-Back tasks produced higher ratings than baseline tasks (Table 5). When comparing sessions, MATB-II was rated as significantly more demanding in the *Pre-Sleep* session than in the *Post* session (medium effect size). A similar pattern was observed for N-Back, where ratings were also higher in the *Pre-Sleep* session, although this difference did not reach statistical significance (small effect size). For both baseline tasks, workload ratings were slightly higher in the *Post-Sleep* session compared to *Pre-Sleep* (negligible to small effect sizes). These differences were negligible for the first baseline but larger for the second baseline, which showed a non-significant increase with a large effect size.

#### 3.2.2 Drowsiness

**Study 1**. MATB-II was associated with the lowest drowsiness ratings overall (Table 6). Across all tasks, the *Easy* session induced greater drowsiness than the *Hard* session. This difference was highly significant for N-Back and SART task (large and medium effect sizes, respectively). MATB-II also showed significantly higher drowsiness rating in the *Easy* session, although the effect was smaller in magnitude (medium effect size). No statistically significant differences were observed for the baseline tasks, where effect sizes ranged from negligible to small.

**Table 6:** Subjective drowsiness statistics.

Study 1 (hard vs easy sessions)						
**Task**	**Easy (M** ±**SD)**	**Hard (M** ±**SD)**	**% Change**	**Test**	***p*** **(Holm)**	**Effect size**
Baseline 1 (pre)	0.33 ± 0.18	0.31 ± 0.19	↓ 4.8%	Paired *t*-test	0.56	*d* = −0.09 (neglible)
MATB-II	0.18 ± 0.15	0.12 ± 0.10	↓ 32.2%	Wilcoxon	0.042^*^	*r* = −0.46 (medium)
N-Back	0.44 ± 0.22	0.19 ± 0.16	↓ 56.1%	Wilcoxon	< 0.001^***^	*r* = −0.86 (large)
SART	0.37 ± 0.23	0.22 ± 0.14	↓ 42.2%	Paired *t*-test	< 0.001^***^	*d* = −0.77 (medium)
Baseline 2 (post)	0.39 ± 0.20	0.34 ± 0.21	↓ 13.3%	Paired *t*-test	0.121	*d* = −0.31 (small)
Study 2 (post-sleep vs. pre-sleep sessions)						
**Task**	**Pre-sleep (M** ±**SD)**	**Post-sleep (M** ±**SD)**	**% Change**	**Test**	***p*** **(Holm)**	**Effect size**
Baseline 1 (pre)	0.28 ± 0.13	0.48 ± 0.20	↑ 42.4%	Paired *t*-test	< 0.001^***^	*d* = 1.18 (large)
MATB-II	0.21 ± 0.12	0.32 ± 0.14	↑ 34.0%	Paired *t*-test	0.001^**^	*d* = 0.80 (large)
N-Back	0.30 ± 0.17	0.43 ± 0.18	↑ 30.8%	Paired *t*-test	0.001^**^	*d* = 0.81 (large)
SART	0.28 ± 0.16	0.40 ± 0.17	↑ 29.4%	Paired *t*-test	0.003^**^	*d* = 0.69 (medium)
Baseline 2 (post)	0.40 ± 0.20	0.50 ± 0.20	↑ 21.1%	Paired *t*-test	0.008^**^	*d* = 0.56 (medium)

↑/↓ indicates direction of change. Significance: ^*^p < 0.05, ^**^p < 0.01, ^***^p < 0.001.

**Study 2**. In line with Study 1, MATB-II task showed the lowest drowsiness ratings overall (Table 6). Across all tasks, subjective drowsiness increased significantly in the *Post-Sleep* session compared to the *Pre-Sleep* session. The largest increase was observed for the first baseline (large effect size), followed by MATB-II and N-Back, which also showed large effects. SART and the second baseline demonstrated significant increases with medium effect sizes.

#### 3.2.3 Mental fatigue

**Study 1**. Across conditions, task sessions showed descriptively higher fatigue than the baseline tasks, with the largest increases observed for the MATB-II, N-Back and SART tasks (Table 7). The *Hard* session of the MATB-II produced significantly greater fatigue than the *Easy* session (large effect size). A similar pattern was found for the N-Back and SART tasks, which also showed significantly higher fatigue in the *Hard* session, although with smaller effect sizes. In contrast, no significant differences were observed for either baseline task. The first baseline showed no change between sessions (negligible effect size), while the second baseline displayed a slight decrease in fatigue during the *Hard* session (small effect size).

**Table 7:** Subjective fatigue statistics.

Study 1 (hard vs easy sessions)						
**Task**	**Easy (M** ±**SD)**	**Hard (M** ±**SD)**	**% Change**	**Test**	***p*** **(Holm)**	**Effect size**
Baseline 1 (pre)	0.23 ± 0.06	0.23 ± 0.07	↑ 2.1%	Wilcoxon	0.877	*r* = 0.02 (negligible)
MATB-II	0.32 ± 0.12	0.40 ± 0.09	↑ 25.2%	Wilcoxon	0.001^**^	*r* = 0.56 (large)
*N*-Back	0.38 ± 0.12	0.44 ± 0.11	↑ 15.6%	Paired *t*-test	0.014^*^	*d* = 0.5 (small)
SART	0.40 ± 0.13	0.45 ± 0.12	↑ 13.9%	Paired *t*-test	0.021^*^	*d* = 0.45 (small)
Baseline 2 (post)	0.27 ± 0.08	0.26 ± 0.10	↓ 4.5%	Wilcoxon	0.418	*r* = −0.2 (small)
Study 2 (post-sleep vs. pre-sleep sessions)						
**Task**	**Pre-sleep (M** ±**SD)**	**Post-sleep (M** ±**SD)**	**% Change**	**Test**	***p*** **(Holm)**	**Effect size**
Baseline 1 (pre)	0.28 ± 0.11	0.36 ± 0.13	↑ 22.0%	Paired *t*-test	0.056	*d* = 0.52 (medium)
MATB-II	0.46 ± 0.09	0.45 ± 0.10	↓ 2.2%	Paired *t*-test	0.502	*d* = −0.13 (negligible)
N-Back	0.53 ± 0.10	0.56 ± 0.09	↑ 4.8%	Paired *t*-test	0.184	*d* = 0.34 (small)
SART	0.52 ± 0.13	0.57 ± 0.10	↑ 8.6%	Wilcoxon	0.065	*r* = 0.48 (medium)
Baseline 2 (post)	0.31 ± 0.09	0.37 ± 0.13	↑ 16.7%	Paired *t*-test	0.056	*d* = 0.53 (medium)

↑/↓ indicates direction of change. Significance: ^*^p < 0.05, ^**^p < 0.01, ^***^p < 0.001.

**Study 2**. Consistent with Study 1, the N-Back and SART tasks elicited the highest fatigue ratings, whereas baseline tasks showed lower overall levels (Table 7). Session effects were generally small and did not reach statistical significance across tasks. Most tasks showed slight increases in fatigue from the *Pre-Sleep* to the *Post-Sleep* session, while MATB-II exhibited a minimal decrease (negligible effect size). The largest increases were observed for the baseline tasks, which approached significance and showed medium effect sizes. N-Back demonstrated a small increase (small effect size), whereas SART showed a larger (medium effect size), though still non-significant, increase.

#### 3.2.4 Mind wandering

**Study 1**. The lowest levels of mind wandering were reported during MATB-II (Table 8). Descriptively, SART scores were higher than those for MATB-II and the first baseline, but lower than the second baseline. Comparisons between *Easy* and *Hard* sessions revealed no statistically significant differences across tasks. Nevertheless, both MATB-II and SART showed medium effect sizes, indicating a tendency toward reduced perceived mind-wandering in the Hard session, whereas baseline tasks showed negligible effects.

**Table 8:** Subjective mind wandering statistics.

Study 1 (hard vs easy sessions)						
**Task**	**Easy (M** ±**SD)**	**Hard (M** ±**SD)**	**% Change**	**Test**	***p*** **(Holm)**	**Effect size**
Baseline 1 (pre)	0.23 ± 0.15	0.26 ± 0.22	↑ 12.5%	Paired *t*-test	0.823	*d* = 0.13 (negligible)
MATB-II	0.10 ± 0.10	0.08 ± 0.10	↓ 18.9%	Wilcoxon	0.381	*r* = −0.41 (medium)
SART	0.25 ± 0.18	0.18 ± 0.14	↓ 26.2%	Wilcoxon	0.381	*r* = −0.30 (medium)
Baseline 2 (post)	0.30 ± 0.24	0.32 ± 0.23	↑ 8.5%	Paired *t*-test	0.823	*d* = 0.13 (negligible)
Study 2 (post-sleep vs. pre-sleep sessions)						
**Task**	**Pre-sleep (M** ±**SD)**	**Post-sleep (M** ±**SD)**	**% Change**	**Test**	***p*** **(Holm)**	**Effect size**
Baseline 1 (pre)	0.21 ± 0.15	0.36 ± 0.20	↑ 40.8%	Paired *t*-test	< 0.001^***^	*d* = 0.92 (large)
MATB-II	0.06 ± 0.11	0.12 ± 0.11	↑ 46.2%	Paired *t*-test	0.118	*d* = 0.38 (small)
SART	0.21 ± 0.15	0.27 ± 0.18	↑ 21.7%	Wilcoxon	0.118	*r* = 0.50 (medium)
Baseline 2 (post)	0.31 ± 0.23	0.41 ± 0.20	↑ 26.0%	Wilcoxon	0.008^**^	*r* = 0.66 (large)

↑/↓ indicates direction of change. Significance: ^*^p < 0.05, ^**^p < 0.01, ^***^p < 0.001.

**Study 2**. Similarly to Study 1, MATB-II elicited the lowest mind wandering ratings overall, with values lower than those observed for the baseline and SART tasks (Table 8). Session effects were more pronounce in this study, with *Post-Sleep* ratings exceeding *Pre-Sleep* ratings across all tasks. The largest increases were observed for the baseline tasks, both of which showed significant *Post-Sleep* elevations (large effect sizes). In contrast, MATB-II and SART also showed increases following sleep, but these differences did not reach statistical significance (small and medium effect sizes, respectively).

### 3.3 Data quality control and anomalies

Data quality checks were conducted across both studies to ensure the reliability of behavioral and subjective measures. Minor issues, such as brief task interruptions or incomplete questionnaire items, were rare and did not affect overall analyses. Particularly, for performance data, a software error resulted in the premature closure of the MATB-II application in four *High* sessions. Since only 1 min of data was lost, these recordings were retained following expert review. As for self-reported questionnaires, overall completion rates were high in both studies (98.6% in Study 1 and 99.4% in Study 2). Given the low proportion of missing responses, participants who were affected by this were retained for the completed questions.

## 4 Discussion

This study investigated whether multiple related operator cognitive states can be jointly elicited and differentiated within a controlled experimental framework using complementary tasks and targeted manipulations. By combining multitasking (MATB-II), working memory (N-back), and sustained attention (SART) paradigms with manipulations of task difficulty and partial sleep restriction, the design aimed to approximate the complexity of operational environments in which cognitive states co-occur and interact. Overall, task difficulty produced robust and consistent effects on both performance and subjective measures, whereas partial sleep restriction primarily influenced subjective states, with more limited and task-dependent effects on performance. These findings support the validity of the experimental design and highlight the importance of combining complementary manipulations to probe different dimensions of cognitive state. Importantly, overlapping patterns across conditions suggest that different manipulations selectively modulate components of shared cognitive and physiological processes, rather than producing uniform effects across states. This partial convergence highlights both the difficulty and necessity of distinguishing cognitive states within a share experimental context and supports the use of multimodal approaches for their characterization.

### 4.1 Effect of task difficulty and partial sleep restriction on performance

In Study 1, increasing task difficulty from *Easy* to *Hard* resulted in performance degradation across tasks. MATB-II was sensitive to level manipulation, showing pronounced effects in tracking error, resource management deviation, and RTs. In addition, faster communication RTs coupled with lower accuracy were observed in the *Hard* session, a pattern consistent with a speed-accuracy trade-off, whereby participants prioritize response speed at the expense of accuracy under increased workload (Dutilh et al., 2011). N-Back showed a comparable response to increased task difficulty, characterized by reduced accuracy and F1 score alongside increases in mean RT, RT variability, error rate, and timeout rate. Together, these results indicate that higher memory load placed greater demands on working memory and executive control, leading to slower and less stable performance, consistent with previous studies (Beh et al., 2021; Ke et al., 2015; Pouliou et al., 2023). Lastly, SART performance exhibited a differentiated response to task difficulty. Error and timeout rates increased in the *Hard* condition, while accuracy, F1 score, and mean RT decreased. The combination of faster responses and increased error rates suggests a shift toward a speed-biased response strategy under higher task demands. This interpretation is consistent with evidence that SART performance is susceptible to speed-accuracy trade-offs, with faster responding systematically associated with increased commission errors (Helton et al., 2009; Seli et al., 2013).

In Study 2, performance differences between *Pre-Sleep* and *Post-Sleep* sessions were smaller and less consistent across tasks. MATB-II showed a trend toward modest performance improvements in the *Post-Sleep* session, whereas N-Back and SART did not show statistically significant differences. Several factors may account for these findings. First, task difficulty was held constant across sessions, limiting the magnitude of performance differences attributable to task demands alone. Second, the 2 h of sleep between sessions may have had opposing effects on performance. On the one hand, this brief period of sleep may have provided some degree of recovery. On the other hand, performing the tests shortly after waking up may have caused sleep inertia, which can temporarily impair cognitive performance (Hursh et al., 2004; Peng et al., 2018). The opposing effects may have partially offset one another, which would have contributed to the absence of clear behavioral differences between the sessions. Other studies have also observed the emergence of compensatory resource allocation following longer periods of sleep deprivation due to awareness of own exhaustion, but these findings may not be directly applicable to the relatively short sleep period in the present study (Hockey et al., 1998; Sullan et al., 2021).

While N-Back performance did not show statistically significant changes, a consistent, mild, trend toward reduced accuracy, increased reaction time, and higher error rates was observed in the *Post-Sleep* session. This consistency with findings of previous N-back research on sleep deprivation, despite the lack of statistical significance in our study, suggests a subtle impact on working memory efficiency that may not have been fully captured by our experimental parameters (Li et al., 2024; Martínez-Cancino et al., 2015). In contrast, SART performance exhibited an opposite trend. In the *Post-Sleep* session, accuracy and F1 score increased, while error and timeout rates decreased, alongside slower RTs and increased RT variability. This pattern may reflect a shift toward a more cautious response strategy, whereby slower responding is associated with improved accuracy (speed-accuracy trade-off). Taken together, these findings suggest that the effects of partial sleep restriction may depend on task demands and response strategies, rather than reflecting a uniform generalized impairment.

### 4.2 Effect of task difficulty and partial sleep restriction on perceived cognitive state

Across both studies, subjective questionnaires revealed clearer and more systematic effects than performance metrics. Subjective mental workload ratings closely tracked task difficulty in Study 1, with MATB-II and N-Back consistently rated as more demanding than baseline conditions and *Hard* sessions eliciting higher workload than *Easy* sessions. In Study 2, *Pre-Sleep* workload ratings were often higher than *Post-Sleep* ratings, which is expected because both sessions involved tasks of equal difficulty and participants may have become more accustomed to the task after a second trial. These results support the effectiveness of the workload manipulation and the use of selected NASA-TLX dimensions as sensitive indicators of perceived task demand.

Subjective drowsiness followed a pattern distinct from workload. In Study 1, *Easy* sessions were associated with higher drowsiness than *Hard* sessions, suggesting that lower stimulation and reduced engagement may promote vigilance decrements. In Study 2, subjective drowsiness increased markedly in the *Post-Sleep* session across all tasks, suggesting that the protocol modulated perceived alertness as intended.

Subjective mental fatigue exhibited more gradual and task-dependent effects. In Study 1, fatigue increased reliably with task difficulty, particularly for N-Back and SART. In Study 2, fatigue changes were more modest, reflecting an increase in perceived fatigue for N-Back and SART tasks but a minimal decrease for MATB-II. Notably, the mental fatigue index was derived from items capturing both perceived mental workload and drowsiness. Therefore, the observed task-dependent differences may reflect differential contributions of workload-related strain and sleepiness-related alertness changes across tasks. Under increased task difficulty (Study 1), fatigue ratings may have been primarily driven by elevated cognitive workload, whereas under partial sleep restriction (Study 2), changes may have been more closely associated with fluctuations in alertness rather than workload. This distinction suggests that subjective fatigue responses may partly depend on the relative balance between cognitive demand and sleep-related factors inherent to each task context.

Subjective mind wandering showed similar trends to drowsiness, with *Easy* and *Post-Sleep* sessions associated with higher mind wandering, particularly during SART and baselines. MATB-II task generally elicited the lowest levels, suggesting its high engagement requirements. While not always statistically significant, this pattern is directionally consistent with prior findings linking reduced alertness to mind wandering (Braboszcz and Delorme, 2011; Zanesco et al., 2024).

### 4.3 Limitations

Several limitations should be acknowledged. First, the participant sample, drawn from specific professional groups may exhibit unique characteristics associated with their professional training and operational experience. While this may limit generalizability, it also provides an opportunity to examine specific response patterns relevant to high-stakes operational contexts. Second, the sample size, particularly in Study 2 (*n* = 27), may have limited sensitivity to detect smaller effects or subtle modulations of performance, contributing to the high number of non-significant findings for performance metrics. Furthermore, in Study 2, the participants performed the same tasks twice on 1 day, so the possible effects of practice or familiarization cannot be completely ruled out. Future studies could address this issue by using a between-subjects design or by including an appropriate control group. Third, the controlled laboratory setting and relatively short task durations may not fully capture prolonged or naturally evolving cognitive states. Finally, subjective measures are inherently susceptible to reporting biases, which should be considered when interpreting discrepancies between subjective and performance results.

### 4.4 Future directions

Future research will leverage this multimodal dataset to develop robust predictive models capable of distinguishing the targeted cognitive states. Integrating physiological signals with the validated behavioral and subjective measures will be essential for improving generalization across tasks and conditions. Such advances are critical for enabling reliable real-time monitoring of cognitive states in operational environments.

### 4.5 Conclusions

The results indicate that task difficulty and partial sleep restriction produce differentiated and partially complementary effects across performance and subjective measures. Specifically, increased task difficulty reduced performance across all tasks, increased perceived mental workload and fatigue, and was associated with lower subjective drowsiness and mind wandering. In contrast, partial sleep restriction produced less consistent changes in task performance and was more clearly associated with increased subjective drowsiness and mind wandering, whereas its effects on mental workload and fatigue were less pronounced and more task-dependent.

Beyond these findings, a central contribution of this work lies in the development and initial validation of a multimodal dataset. By combining complementary tasks and targeted manipulations within a consistent experimental setting, the dataset supports the joint investigation of multiple interacting cognitive states, providing a closer approximation to operational environments in which such states coexist and evolve dynamically. This dataset lays the groundwork for future multimodal modeling, supporting the integration of physiological, behavioral, and subjective data to improve the detection and differentiation of cognitive states. Ultimately, these findings provide a basis for future work toward the development of robust, real-time monitoring systems for high-demand operational contexts.

## Figures and Tables

**Figure:**
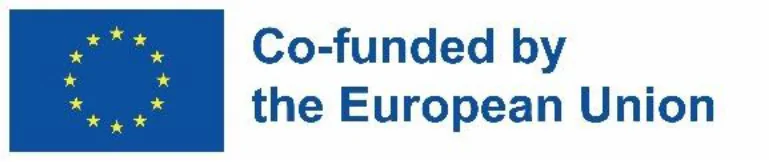


## Data Availability

The datasets presented in this article are not readily available because they are subject to confidentiality and security restrictions arising from this work's participation in a European defense-related research framework. Access to the dataset is limited by the project agreement, applicable regulatory requirements, and the need to protect sensitive information. Consequently, the dataset cannot be publicly released or shared. Nevertheless, the proposed framework, methodology, and experimental procedures are described in sufficient detail to support scientific transparency and reproducibility within the constraints of these confidentiality obligations.
