# Supplementary material for: Differentiating linked cognitive states under task demand and sleep restriction: toward an experimental framework for operational monitoring
Source: Front Neuroergon. 2026 Oct 6;7:1909914. doi: 10.3389/fnrgo.2026.1909914 (PMC13680395; doi:10.3389/fnrgo.2026.1909914)
Supplement: Supplementary file 1 [file Table_1.docx]

***Supplementary Material***

# 1 Supplementary Figures

**Supplementary Figure 1.** Subjective Mental Workload Assessment Distribution Across Tasks and Sessions for Study 1

Supplementary Figure 2. Subjective Mental Workload Assessment Distribution Across Tasks and Sessions for Study 2

Supplementary Figure 3. *Subjective Drowsiness Assessment Distribution Across Tasks and Sessions for Study 1*

Supplementary Figure 4. Subjective Drowsiness Assessment Distribution Across Tasks and Sessions for Study 2

Supplementary Figure 5. Subjective Fatigue Assessment Distribution Across Tasks and Sessions for Study 1

Supplementary Figure 6. Subjective Fatigue Assessment Distribution Across Tasks and Sessions for Study 2

Supplementary Figure 7. Subjective Mind Wandering Assessment Distribution Across Tasks and Sessions for Study 1

**Supplementary Figure 8.** Subjective Mind Wandering Assessment Distribution Across Tasks and Sessions for Study 2
